# Hypoxia Disruption of Vertebrate CNS Pathfinding through EphrinB2 Is Rescued by Magnesium

**DOI:** 10.1371/journal.pgen.1002638

**Published:** 2012-04-12

**Authors:** Tamara J. Stevenson, Tony Trinh, Cory Kogelschatz, Esther Fujimoto, Mark E. Lush, Tatjana Piotrowski, Cameron J. Brimley, Joshua L. Bonkowsky

**Affiliations:** 1 Division of Pediatric Neurology, Department of Pediatrics, University of Utah School of Medicine, Salt Lake City, Utah, United States of America; 2 University of Utah School of Medicine, Salt Lake City, Utah, United States of America; 3 Department of Neurobiology and Anatomy, University of Utah School of Medicine, Salt Lake City, Utah, United States of America; 4 Stowers Institute for Medical Research, Kansas City, Missouri, United States of America; Stanford University School of Medicine, United States of America

## Abstract

The mechanisms of hypoxic injury to the developing human brain are poorly understood, despite being a major cause of chronic neurodevelopmental impairments. Recent work in the invertebrate *Caenorhabditis elegans* has shown that hypoxia causes discrete axon pathfinding errors in certain interneurons and motorneurons. However, it is unknown whether developmental hypoxia would have similar effects in a vertebrate nervous system. We have found that developmental hypoxic injury disrupts pathfinding of forebrain neurons in zebrafish (*Danio rerio*), leading to errors in which commissural axons fail to cross the midline. The pathfinding defects result from activation of the hypoxia-inducible transcription factor (*hif1*) pathway and are mimicked by chemical inducers of the *hif1* pathway or by expression of constitutively active *hif1α*. Further, we found that blocking transcriptional activation by *hif1α* helped prevent the guidance defects. We identified *ephrinB2a* as a target of *hif1* pathway activation, showed that knock-down of *ephrinB2a* rescued the guidance errors, and showed that the receptor *ephA4a* is expressed in a pattern complementary to the misrouting axons. By targeting a constitutively active form of *ephrinB2a* to specific neurons, we found that *ephrinB2a* mediates the pathfinding errors via a reverse-signaling mechanism. Finally, magnesium sulfate, used to improve neurodevelopmental outcomes in preterm births, protects against pathfinding errors by preventing upregulation of *ephrinB2a*. These results demonstrate that evolutionarily conserved genetic pathways regulate connectivity changes in the CNS in response to hypoxia, and they support a potential neuroprotective role for magnesium.

## Author Summary

How hypoxia damages the developing human brain is poorly understood, despite being a major cause of life-long neurologic and psychiatric problems. Premature infants are especially at risk for these problems, with increased rates of attention-deficit disorder, autism, cerebral palsy, epilepsy, psychiatric disorders, and cognitive impairment. It is unknown whether hypoxia can cause errors in the connections of neurons in the vertebrate nervous system. We used zebrafish, a vertebrate model animal, to answer this question. We found that hypoxic injury causes errors in how neurons connect. We went on to determine that a specific genetic pathway, the *hif1* pathway, is activated by hypoxia and turns on downstream genes, which cause the connection problems. One of the genes activated by *hif1*, *ephrinB2a*, is responsible for many of the connection problems. Importantly, magnesium, used as a treatment for some preterm births, is able to help protect against the neuron connection errors. Our results show that hypoxia in vertebrates does cause errors in neuron connections and that magnesium can help prevent this.

## Introduction

Hypoxic injury to the developing human brain is a major cause of both acute and chronic neurodevelopmental impairments. Premature infants, particularly those characterized by very-low birth weights (VLBW; less than 1,500 g) are the population at greatest risk for chronic hypoxic injury and for adverse neurocognitive outcomes [1]. Causes of hypoxia in these infants include chronic lung disease, pulmonary hypertension, congenital heart disease, and placental insufficiency. Up to 35% of VLBW infants experience neurodevelopmental impairments including attention-deficit disorder, autism, cerebral palsy, epilepsy, psychiatric disorders, and mental retardation/cognitive impairment [2]–[5]. While survival rates have improved dramatically for premature infants, neurodevelopmental outcomes have not [6], [7]; in fact, the total number of VLBW infants has increased over the past decade [8].

Strategies to protect against the effects of prematurity and chronic hypoxic injury to the central nervous system (CNS) have been limited since the pathophysiology that leads to the adverse neurodevelopmental outcomes is uncertain. Indirect measures in humans have shown altered connectivity in the brains of children born prematurely [9], [10], and the period between 20 weeks gestation and term birth (40 weeks gestation) is a period of extensive commissural and projection axon extension [11]. Most VLBW infants are born between 23 and 28 weeks gestation, and both pre-term and term infants experience episodic, often unrecognized, hypoxemia [12].

The mechanisms by which hypoxia disrupts connectivity in vertebrates are not known. Recent work in the invertebrate *C. elegans* has shown that hypoxia causes discrete axon pathfinding errors in certain interneurons and motorneurons by increased expression of the Eph receptor *vab-1* [13]. However, it is unknown whether similar pathfinding errors, and similar genetic pathways and molecular mechanism, also occur in the more complex vertebrate CNS.

We hypothesized that hypoxia might specifically affect axon pathfinding and the development of CNS connectivity in vertebrates. To test this we developed a zebrafish (*Danio rerio*) model for investigating hypoxia at different stages of CNS development, and generated transgenic lines to test the genetic pathways involved. We analyzed both genetic and chemical modifiers of the molecular response of hypoxia on pathfinding, including cloning zebrafish *hif1α* and generating a constitutively active form of it. We found that hypoxia disrupts axon pathfinding in vertebrates through an evolutionarily conserved mechanism, by activation of the *hif1α* pathway and increased expression of *ephrinB2*. Further, we tested the role of magnesium sulfate, which is known to improve neurodevelopmental outcomes when given to mothers of infants at risk for premature delivery [14]. We found that magnesium sulfate helps reduce hypoxia-induced upregulation of *ephrinB2*, and decreases the frequency of pathfinding errors.

## Results

We developed a system in zebrafish to examine the effects of hypoxia on the development of CNS connectivity. We used a small, airtight plexiglass chamber in which zebrafish embryos were placed at different developmental stages. Hypoxia was induced by use of a digital controller that regulated nitrogen gas flow, and we pre-equilibrated solutions to either normoxia or hypoxia for at least 4 hours before use. Previous reports have shown that early zebrafish embryos tolerate anoxia, especially before 24 hours post-fertilization (hpf), but the anoxia can slow development [15], [16]. Because we wanted to model hypoxic insults to CNS development, we tested a wide range of hypoxic conditions at different times to determine the degree of hypoxia that embryos could tolerate with minimal mortality (Table 1). We assessed mortality at 72 hpf, at which point major stages of CNS development have occurred, including neurogenesis, cell-type specification, axon pathfinding, and synaptogenesis, following either 12 or 24 hour periods of hypoxia, using at least 30 embryos per condition. We found that zebrafish embryos can tolerate long periods of stringent hypoxia, especially at earlier developmental stages. Because we noted dysmorphic development in embryos exposed to 0.5% hypoxia (0.5% pO_2_) or lower, we used 1% hypoxia (1% pO_2_; normoxia is 21% pO_2_) for experiments.

**Table 1:** Hypoxia and survival.

Hypoxia conditions (% pO_2_)	Timing of hypoxia (hpf)	Survival (% alive at 72 hpf)
normoxia (21%)	n/a	100%
8%	24–48 hpf	100%
8%	48–72	100%
2%	24–48	100%
2%	48–72	100%
1%	24–48	100%
1%	48–72	0%

Table of survival percentages following hypoxia exposure in embryonic zebrafish at different ages. 30 or more embryos were examined for each time-point.

### Developmental hypoxia causes axon pathfinding errors

**Figure 1:**
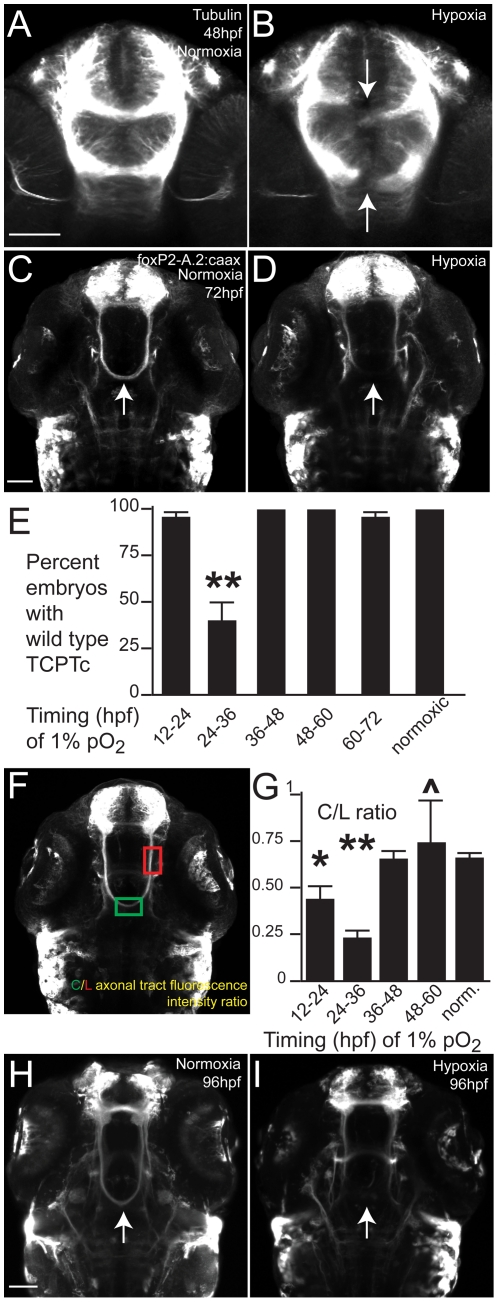
Hypoxia disrupts axon pathfinding in the developing CNS. Confocal images are maximum intensity projections of whole-mount embryos, ventral views, rostral top, scale bars 50 µm. (A, B) 48 hpf embryos, acetylated tubulin immunohistochemistry. (B) Following 1% hypoxia (1% pO_2_) from 24–36 hpf, anterior commissure and post-optic commissural tracts are disrupted (arrows). (C–I) Tg(*foxP2-enhancerA.2:egfp-caax*) embryos (abbreviated *foxP2-A.2:caax*), GFP immunohistochemistry. (C) Normoxic embryo at 72 hpf; TCPT commissure (TCPTc) (arrow). (D) Embryo following hypoxia from 24–36 hpf; TCPTc is absent (arrow). (E) Graph showing percent Tg(*foxP2-A.2:caax*) embryos with wild-type TCPTc following hypoxia exposure at different ages. Error bars standard error of the proportion; ** *p*<0.0001. (F) C/L ratio determination. Confocal image of 72 hpf embryos illustrating determination of ratio of commissure intensity to intensity of longitudinal axons. (G) Quantification of TCPTc errors in Tg(*foxP2-A.2:caax*) embryos following hypoxia exposure at different ages. C/L (commissure:longitudinal) intensity ratio quantification along *y*-axis. *p<0.05; ** *p*<0.0001. ∧mark indicates significant mortality at this stage. Errors bars standard error of the mean. (H) Normoxic embryo at 96 hpf; TCPT is intact (arrow). (I) Embryo at 96 hpf following 1% hypoxia from 24–36 hpf; TCPTc is absent (arrow).

To visualize effects of hypoxia on pathfinding, we evaluated several different transgenic lines as well as a pan-axonal antibody against acetylated tubulin. Using anti-acetylated tubulin, we found at low frequency subtle pathfinding errors. Further, in less than 5% of embryos we found severe axon pathfinding errors, most often of commissural axon tracts (Figure 1A, 1B). However, because of the low frequency of severe errors and difficulty visualizing and quantifying the subtle errors using anti-acetylated tubulin, we generated a transgenic line in which a small subset of axons expressed membrane-targeted GFP: Tg(*foxP2-enhancerA.2:egfp-caax*). This line precisely labeled a few distinct neuron types, with retinal, commissural, and longitudinal axons [17] (Figure 1C).

We examined axon tracts in Tg(*foxP2-enhancerA.2:egfp-caax*) embryos following 12-hour periods of hypoxia, and found a reproducible defect in formation of the tract of the commissure of the posterior tuberculum (TCPT) [18]. This defect was never seen in wild-type embryos (Figure 1D), and there was no overall difference in fluorescence intensities of normoxic compared to hypoxic transgenic embryos. Following hypoxia, the TCPT commissure (TCPTc) either did not form, or had fewer axons crossing.

We noted significant variability in the severity of the phenotype, with some embryos lacking the TCPTc, some with a normal appearance to the TCPTc, and some embryos with intermediate phenotypes. The intermediate phenotypes were embryos in which the number of TCPTc axons was reduced. The percentage of embryos with loss or near-complete absence of the TCPTc following 1% pO_2_ from 24–36 hpf was 60% (Figure 1E), with no significant effects of hypoxia when examined at other time-points. Because of the potential for subjectivity in scoring intermediate phenotypes of disrupted TCPTc, and in order to increase our ability to detect more subtle changes in the number of TCPTc axons crossing, we developed a quantitative measure of TCPTc axon crossing. We compared fluorescence intensity ratios of commissural to longitudinal axons of the TCPT axons, in normoxic versus hypoxic embryos (C/L ratio; Figure 1F; Methods). The average ratio for normoxic embryos was 0.639, whereas for hypoxic embryos (1% pO_2_ from 24–36 hpf) the average was statistically different at 0.225 (*p*<0.0001 for two-tailed *t* test) (Table 2). Thus, a decrease in the C/L ratio represents a decrease in the number of axons crossing in the TCPTc.

**Table 2:** Summary of results for experiments involving C/L ratios.

Genotype	Variable 1	Variable 2	Variable 3	C/L Average	C/L St. Dev.	C/L SEM	C/L 95% CI	*p*
foxA.2:egfpcaax	normoxia			0.639	0.122	0.0354	0.15585	reference
foxA.2:egfpcaax	hypoxia	12–24 hpf		0.412	0.24	0.091	0.443	0.0095
foxA.2:egfpcaax	hypoxia	24–36 hpf		0.225	0.193	0.0421	0.0826	0.0001
foxA.2:egfpcaax	hypoxia	36–48 hpf		0.612	0.233	0.0487	0.0789	0.6522
foxA.2:egfpcaax	hypoxia	48–60 hpf		0.719	0.546	0.273	0.536	0.5524
foxA.2:egfpcaax	hypoxia	60–72 hpf		0.956	0.9	0.519	1.1081	0.0975
foxA.2:egfpcaax	normoxia			0.674	0.237	0.0632	0.0928	reference
foxA.2:egfpcaax	normoxia	100 mMDMOG		0.503	0.584	0.162	0.458	0.3915
foxA.2:egfpcaax	normoxia	250 mMDMOG		0.428	0.175	0.0619	0.15	0.0163
foxA.2:egfpcaax	normoxia	500 mMDMOG		0.284	0.214	0.0713	0.329	0.0015
foxA.2:egfpcaax	hypoxia			0.594	0.156	0.034	0.0665	reference
foxA.2:egfpcaax	hypoxia	20 mcM CAY10585		0.712	0.19	0.044	0.0854	0.03
elavl3:Gal4; FoxA.2caax; UAS:hif1[mut]	normoxia			0.403	0.137	0.028	0.055	reference
elavl3:Gal4; FoxA.2caax; UAS:hif1[mut]	normoxia	efn morpholino		0.633	0.134	0.0506	0.0992	0.0005
elavl3:Gal4; FoxA.2caax; UAS:hif1	normoxia			0.729	0.291	0.092	0.18	0.0001
elavl3:Gal4; FoxA.2caax; UAS:hif1[mut]	normoxia	250 mM Mg		0.346	0.141	0.04252	0.05527	0.2619
foxA.2:egfpcaax	hypoxia	uninjected		0.274	0.0867	0.0119	0.0233	reference
foxA.2:egfpcaax	hypoxia	efn morpholino		0.481	0.18	0.0229	0.0449	0.0001
foxA.2:egfpcaax	normoxia	uninjected		0.339	0.07	0.023	0.0458	reference
foxA.2:egfpcaax	normoxia	efn morpholino		0.286	0.066	0.018	0.0344	0.0823
foxA.2:Gal4; foxA.2:egfpcaax	hypoxia	injected	UAS:efn	0.548	0.313	0.0809	0.158	0.351
foxA.2:Gal4; foxA.2:egfpcaax	hypoxia	injected	UAS:efnΔc	0.745	0.207	0.0822	0.161	0.0006
foxA.2:Gal4; foxA.2:egfpcaax	hypoxia	uninjected		0.473	0.265	0.0531	0.104	reference
foxA.2:Gal4; foxA.2:egfpcaax	normoxia	injected	UAS:efn	0.572	0.265	0.0944	0.185	0.3459
foxA.2:Gal4; foxA.2:egfpcaax	normoxia	injected	UAS:efnΔc	0.649	0.266	0.0897	0.18	0.7809
foxA.2:Gal4; foxA.2:egfpcaax	normoxia	uninjected		0.682	0.302	0.0675	0.283	reference
foxA.2:egfpcaax	hypoxia			0.172	0.07244	0.01811	0.0284	reference
foxA.2:egfpcaax	hypoxia	25 mM Mg		0.321	0.14292	0.04519	0.05602	0.0017
foxA.2:egfpcaax	hypoxia	100 mM Mg		0.398	0.14426	0.04562	0.0617	0.0001
foxA.2:egfpcaax	hypoxia	250 mM Mg		0.451	0.16983	0.05661	0.09424	0.0001
foxA.2:egfpcaax	normoxia	500 mcM DMOG		0.624	0.225	0.047	0.0918	reference
foxA.2:egfpcaax	normoxia	500 mcM DMOG	100 mM Mg	0.907	0.508	0.106	0.203	0.0187

C/L axonal tract fluorescence intensity ratio analysis comparing commissural axons to longitudinal axons of embryos for the different experiments reported in this paper. Genotype is listed in the left-hand column; conditions for the experiment are listed in middle columns, and results (C/L ratios including average, standard deviation, standard error of the mean, and 95% confidence interval) are shown in the right-hand columns, as well as the two-tailed *t* test *p* value in the far right column.

We examined the effects of hypoxia exposure at different developmental stages (Figure 1G), with at least 24 embryos for each period, and analysis at 72 hpf. We found that hypoxia during 24 36 hpf disrupted TCPTc formation, as shown by the statistically significant decrease in the C/L ratio. Increased duration of hypoxia up to 36 hours did not worsen the C/L ratio. Further, when we analyzed the TCPTc at 96 hpf, following hypoxia from 24–36 hpf, there was persistent failure of TCPTc crossing (Figure 1H, 1I). Our hypoxia conditions for 12 hours were therefore followed by either 36 or 60 hours of recovery in normoxia. This demonstrates that the pathfinding errors are not due to a simple maturational delay in axon extension, and that the TCPT axons do not then re-cross the midline.

**Figure 2:**
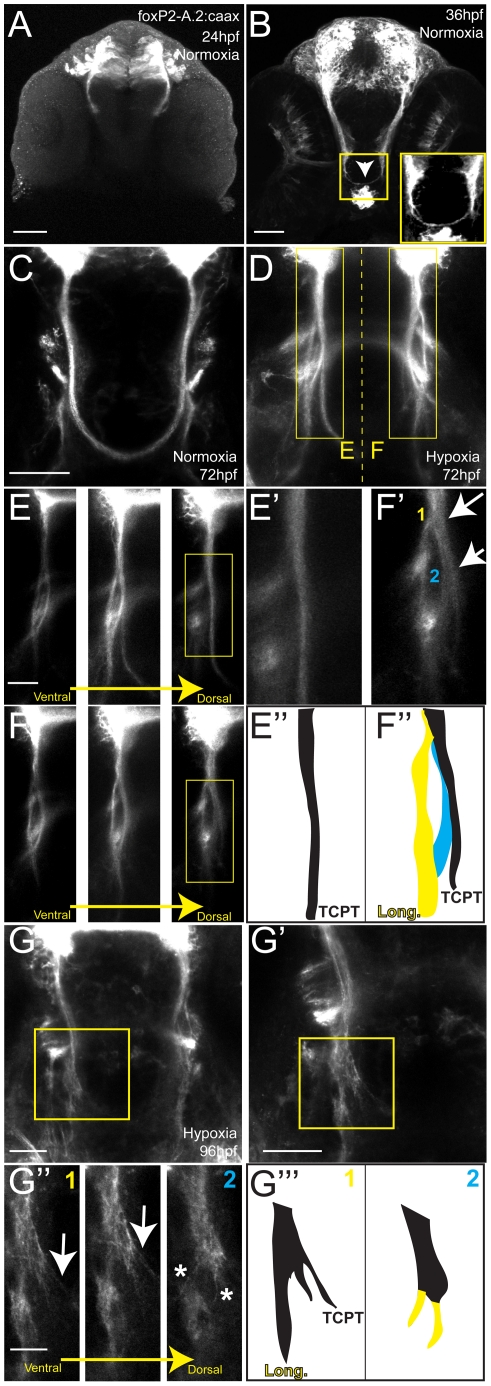
Hypoxia acts during development of the TCPTc by disrupting axon pathfinding. Confocal whole-mount images, anti-GFP immunohistochemistry, of Tg(*foxP2-A.2:caax*) embryos, ventral views, rostral top, except as noted images are maximum intensity projections, scale bars 50 µm except E–E′ and H′ 25 µm; H″ 12.5 µm. (A) Embryo at 24 hpf, cell bodies and initial axon processes are visible. (B) Embryo at 36 hpf; TCPTc has formed (arrowhead, magnified in inset). (C) TCPTc has formed completely by 72 hpf in normoxia. (D) Disruption of TCPTc in hypoxia. Dotted line shows the subsequent high-resolution pictures from the inset boxes: E–E″ from the left hand TCPTc with normal pathfinding; F–F″ with the disrupted TCPTc. (E) Higher magnification views of embryo at 72 hpf, single confocal slices, showing decussation of TCPT commissural and longitudinal axon tracts at different dorsal-ventral levels. (E′) Inset from dorsal-most level, single confocal slice, shows TCPTc arises as a single axon tract. (E″) Schematic of (E′) showing the single tract that will give rise to the TCPTc. (F) Mirror-images of the disrupted TCPTc from the right-hand of image (D), single confocal slices. The dorsal-most image shows the disrupted axon pathfinding. (F′) High-resolution single confocal slice, shows the two aberrant axon pathfinding errors made, 1 and 2, as axons leave the TCPT to join a longitudinal tract. (F″) Schematic of (F′) illustrating the aberrant pathfinding of axons leaving the TCPTc. (G) Hypoxic embryo at 96 hpf and higher resolution shown in (G′). (G″) is high-resolution ventral-to-dorsal single confocal slices. Arrows point to sparse axons giving rise to the TCPTc. Asterisks demonstrate axons aberrantly turning caudally and failing to join the TCPTc in the dorsal-most image. (G′″) Schematic of (G″) illustrating the aberrant pathfinding of axons leaving the TCPTc at different ventral-dorsal levels.

To determine whether the observed pathfinding phenotype following hypoxia correlates with the timing of TCPTc formation, we examined normal development of TCPT axons. The first axons project by 24 hpf (Figure 2A), and by 36 hpf axons are crossing the midline and forming the TCPTc (Figure 2B). Therefore, our observation that maximal effects of hypoxia occur when embryos are exposed from 24 to 36 hpf is consistent with the timing of axon pathfinding.

We then examined the fate of the aberrant axons following hypoxia. Normally, the TCPT axons in Tg(*foxP2-enhancerA.2:egfp-caax*) embryos split (Figure 2C) into commissural and longitudinal portions, with the majority of axons crossing. In contrast, following hypoxia, most axons fail to cross the midline and turn to aberrantly follow a longitudinal pathway (Figure 2D). High-resolution pictures of the TCPTc following hypoxia (Figure 2E–2G′″) show that the TCPTc axons destined to cross the midline arise dorsally and remain tightly bundled as they cross the midline (Figure 2E–2E″). In contrast, following hypoxia, the TCPT axons split, with some crossing the TCPTc, whereas others turn caudally and join the longitudinal, more ventral tracts (Figure 2F–2G′″). Thus, the TCPTc hypoxia phenotype is characterized by axons specifically turning to follow an erroneous pathway.

**Figure 3:**
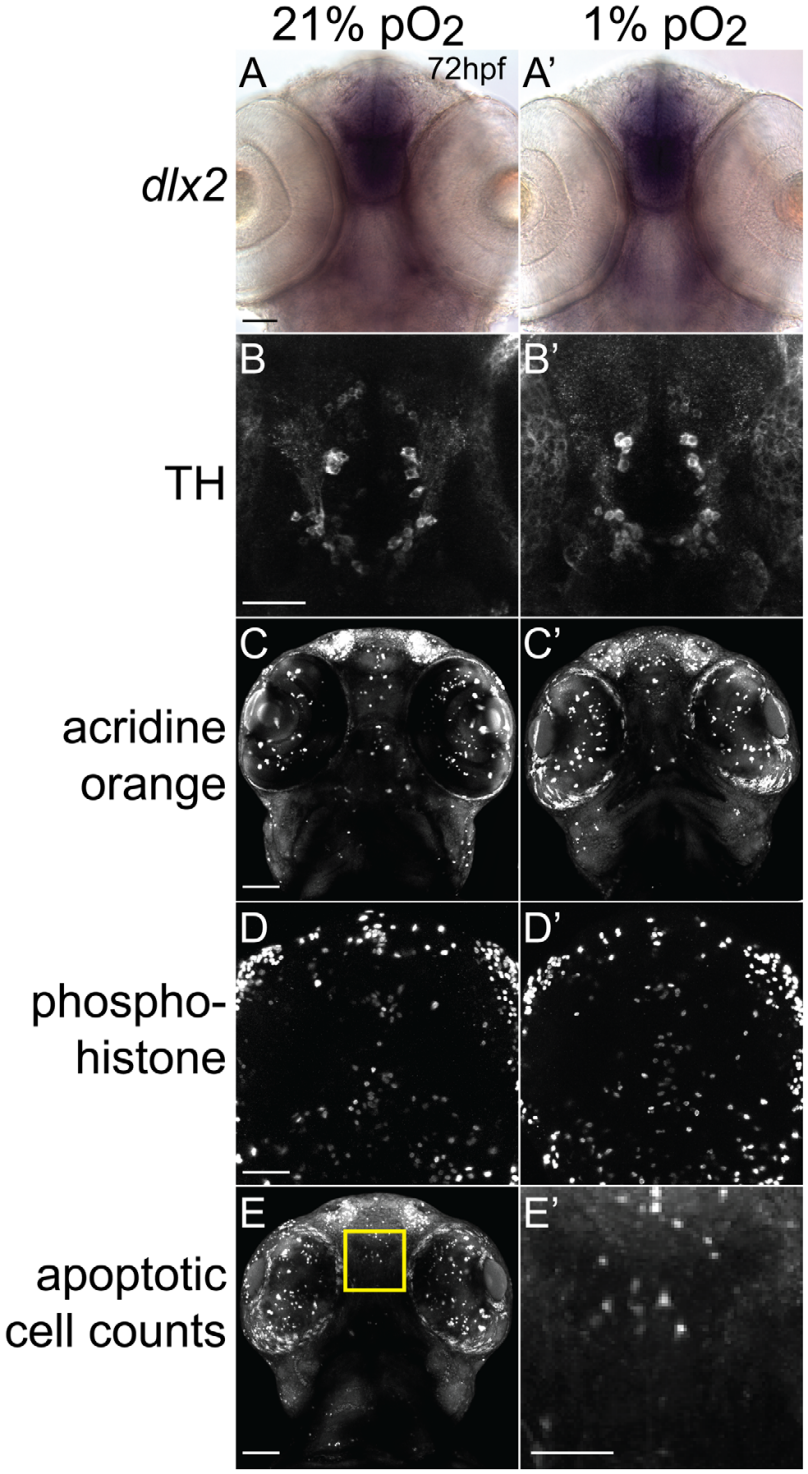
Developmental hypoxia does not affect general CNS development. Ventral views, rostral to the top, scale bar 50 µm (except E′, 25 µm). Whole-mount embryos, shown as brightfield (A), or confocal maximum intensity z-projections (B–E). (A, A′) *in situ* hybridization for *dlx2* shows no difference in pattern. (B, B′) α-tyrosine hydroxylase (TH) antibody staining pattern is unaffected. (C, C′) Acridine orange shows similar numbers of apoptotic cells. (D, D′) Phospho-histone H3 antibody staining shows similar numbers of mitotic cells. (E) Confocal image of acridine orange stain demonstrating region for determining apoptotic cell counts. Inset shows high-magnification (E′) for counting cells in the 100 µm×100 µm area.

Possible explanations for the observed disruption in axon pathfinding could be a general effect on CNS patterning; on neuron specification; or on apoptosis or cellular proliferation. To address these possibilities, we compared hypoxic embryos to normoxic embryos using a variety of cellular markers (Figure 3). We did not find any significant changes in a variety of markers, including *dlx2* for CNS forebrain and diencephalon patterning, TH antibody staining for cell-type specification of *tyrosine hydroxylase*-expressing neurons, acridine orange for apoptosis, and anti-phosphohistone H3 antibody for proliferation. We quantified apoptosis at 72 hpf in a region of the telencephalon that includes TCPT neuron cell bodies, and found no significant difference between normoxic and hypoxic embryos (cell counts 6.5 in normoxia, 7.1 in hypoxia, standard deviation 4.6, n = 30 embryos, *p* = 0.6; Figure 3; Methods).

### Hif1α mediates hypoxia's effects on pathfinding

**Figure 4:**
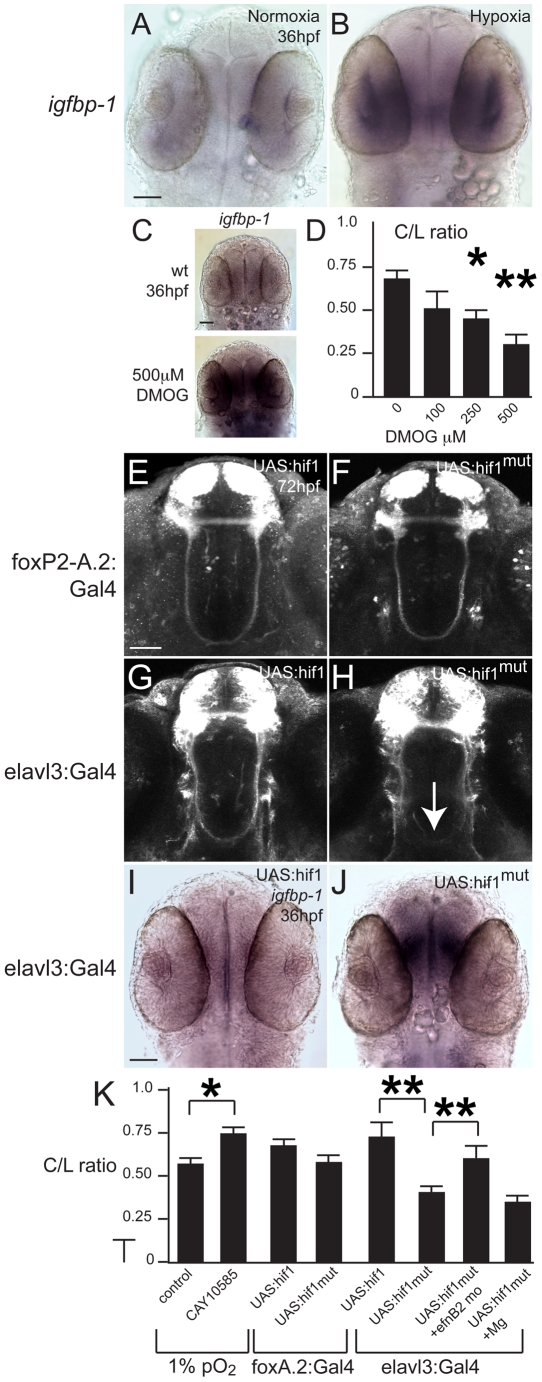
Developmental hypoxia induces the *hif1* pathway and disrupts pathfinding via a non-cell autonomous mechanism. Whole-mount embryos, 36 hpf, ventral views, rostral to the top, scale bar 50 µm. Images are brightfield views. (A, B) Staining for the *igfbp-1* gene activated by hypoxia shows that hypoxia effectively induces the *hif1* pathway. (C) DMOG activates the *hif1* pathway, as shown by increased *igfbp-1* expression at 36 hpf following DMOG exposure from 24–36 hpf. (D) Increasing DMOG concentration from 24–36 hpf leads to increased rates of TCPTc pathfinding errors, assayed by C/L ratios. *p<0.05; ** *p*<0.005. Error bars SEM. (E–H) Confocal images, 72 hpf, ventral views, rostral top. (E, F) Normal TCPT pathfinding when *hif1α* or activated *hif1α* ^mut^ is expressed in *foxP2* neurons. (G, H) Expression of *hif1α* ^mut^, but not *hif1α*, disrupts TCPT pathfinding when expressed in neighboring neurons. (I, J) Expression of *hif1α* ^mut^, but not *hif1α*, leads to activation of the hypoxia pathway as assayed by *igfbp-1* expression. (K) C/L intensity ratios of experiments. *p<0.05; ** *p*<0.005. Error bars SEM.

Cellular responses to hypoxia are coordinated by activation of the *hif1* pathway [19]. *hif1α* is a basic helix-loop-helix transcription factor ubiquitously expressed, but which is normally hydroxylated and degraded under normoxic conditions. In hypoxia *hif1α* hydroxylation is inhibited, and *hif1α* is able to activate a downstream genetic pathway of target genes that modify an organism's response to hypoxia, for example, by increased angiogenesis, [20]. We wished to determine whether *hif1* pathway activation was mediating the TCPTc pathfinding errors. First, we wanted to establish whether *hif1* pathway activation was occurring from our hypoxia model. We decided to examine expression of *igfbp-1*, a known downstream transcriptional target of *hif1* pathway activation from hypoxia in vertebrates, including zebrafish and humans [21]–[24]. Following hypoxia from 24–36 hpf, *igfbp-1* expression was increased (Figure 4A, 4B). To demonstrate a role for *hif1α*, we used dimethyloxaloglycine (DMOG) to activate *hif1α* by inhibition of prolyl hydroxylase or factor inhibiting hypoxia-inducible factor [25], in the absence of hypoxia. Normoxic embryos were exposed to varying DMOG amounts from 24–36 hpf. Increasing amounts of DMOG led to increasing expression of *igfbp-1* (Figure 4C); and increased pathfinding errors of the TCPTc (n>26 embryos for all conditions) (Figure 4D; Table 2). Further, an inhibitor of *hif1α* transcription CAY10585 [26] was able to reduce the C/L ratio in hypoxia (Figure 4K). These results suggest that the TCPT commissure errors due to hypoxia are caused by activation of the *hif1* pathway.

### Pathfinding errors are cell-intrinsic to the effects of hypoxia

To determine whether *hif1α*'s role on the TCPT commissure is cell-autonomous, we decided to misexpress *hif1α* or *hif1α* ^mut^, a constitutively active form of *hif1α*, using the UAS/GAL4 system [27]. We cloned the zebrafish *hif1α* cDNA, and then made *hif1α* ^mut^ by changing proline 621 to alanine in the conserved LXXLAP motif of HIF-1α [28]. We generated stable transgenic lines expressing *hif1α* or *hif1α* ^mut^ downstream of UAS, with a viral 2A peptide fused to GFP-caax or RFP-caax to generate a bicistronic message [29]. The use of the bicistronic message allowed us to monitor whether expression of *hif1α* or *hif1α* ^mut^ was occurring from the UAS, by the presence of fluorophore expression. We drove expression either in the TCPT neurons using Tg(*foxP2-enhancerA.2:Gal4-VP16*), or pan-neuronally using Tg(*elavl3:Gal4-VP16*) (Figure 4E–4K). We did not observe any TCPTc pathfinding errors when we expressed either *hif1α* or *hif1α* ^mut^ in the TCPT neurons (n>30 embryos for each genotype). In contrast, when we expressed *hif1α* ^mut^ but not *hif1α* using Tg(*elavl3:Gal4*), we observed TCPTc errors (Figure 4G, 4H). For both *hif1α* and *hif1α* ^mut^ we screened and isolated two different independent UAS transgenic alleles each, and examined fluorescence expression when crossed to a Gal4-driver line; all subsequent experiments were then based on use of a single allele with robust expression. Quantification of C/L ratios in Tg(*elavl3:Gal4*); Tg(*foxP2-enhancerA.2:egfpcaax*); Tg(*UAS: hif1α* ^mut^ *-2A-TagRFP*) embryos (n = 25 embryos) compared to Tg(*elavl3:Gal4*); Tg(*foxP2-enhancerA.2:egfpcaax*); Tg(*UAS:hif1α-2A-TagRFP*) embryos (n = 10) was statistically significant (Figure 4K). We confirmed that the hypoxia pathway is activated by expression of *hif1α* ^mut^ by showing that *igfbp-1* is up-regulated (Figure 4I, 4J).

To try to further localize the site of action of hypoxia pathway activation, we expressed *hif1α* ^mut^ in neurons neighboring the TCPT axons. We crossed Tg(*otpb.A:Gal4-VP16*) [30] to Tg(*foxP2-enhancerA.2:egfpcaax*), and injected embryos with a plasmid carrying *UAS:hif1α^mut^-2A-TagRFP*. In these embryos *hif1α* ^mut^ is expressed neighboring the TCPT axons as they extend longitudinally, prior to decussating. We did not observe any defects in formation of the TCPTc (0%, n = 50 embryos, Figure S1). While we can not exclude the possibility that levels of *hif1α* ^mut^ were insufficient in these experiments to disrupt pathfinding, we think it is more likely that (if a suitable Gal4 line were available) expression targeted to the CNS midline and/or at the site of the decussation would be effective. This is based on the expression pattern of a potential receptor guiding TCPTc axons (below, FFure 6). Thus, the pathfinding errors in TCPT axons resulting from hypoxia can be mimicked by activation of the *hif1* pathway pan-neuronally, but not in TCPT neurons alone.

### Hypoxia causes pathfinding errors by upregulation of ephrinB2a

Our finding that *hif1* pathway activation has a non-cell-autonomous effect on TCPT axon pathfinding suggested that hypoxia affected pathfinding through effects on cell-cell signaling. The Eph-ephrin signaling system has conserved roles in axon pathfinding in both vertebrates and invertebrates and controls aspects of commissural axon pathfinding [31], [32]. Further, in *C. elegans* hypoxia upregulates the ephrin receptor *vab-1*, while knockdown of its ligand *efn-2* prevents hypoxia pathfinding defects [13]. *ephrinB2a* is the zebrafish gene with greatest sequence conservation to *efn-2* and further is expressed in telencephalic neurons in the zebrafish embryonic CNS [33].

**Figure 5:**
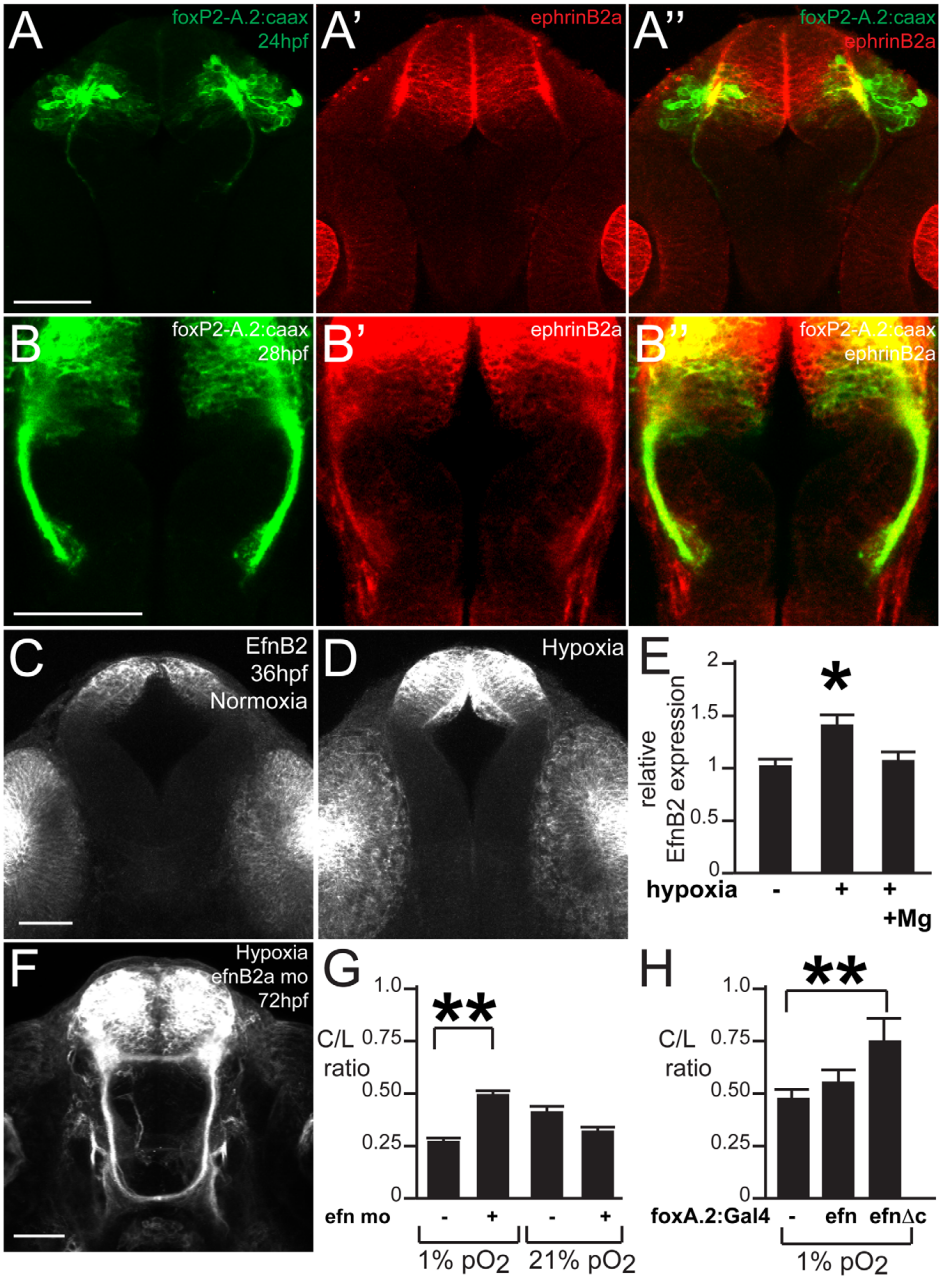
ephrinB2a mediates the hypoxia-induced TCPT pathfinding errors. (A–B) ephrinB2a is expressed in TCPT neurons. Confocal maximum intensity projections of whole-mount embryos, double immunohistochemistry for GFP and EfnB2a, ventral views, rostral top, scale bar 50 µm. (A–A″) TCPT neurons express ephrinB2a as they begin to extend axons. (B–B″) TCPT commissural axons express ephrinB2a as they cross the midline. (C–D) Hypoxia leads to increased expression of EfnB2a. Maximum intensity projections of 36 hpf embryos, ventral views, rostral to the top, EfnB2a immunohistochemistry. (E) Quantification of EfnB2a levels, normalized to normoxia. *p<0.05. Error bars SEM. (F) Knockdown of EfnB2a expression with morpholino rescues TCPTc pathfinding. Tg(*foxP2-A.2:caax*) embryo at 72 hpf; confocal maximum intensity projection, ventral view, rostral top, scale bar 50 µm. (G) C/L intensity ratio quantifications show ephrin morpholino rescue of hypoxia pathfinding errors. *p<0.01; ** *p*<0.001. Error bars SEM. (H) C/L intensity ratios show *UAS:ephrinΔc* rescues hypoxia pathfinding errors. ** *p*<0.001. Error bars SEM.

**Figure 6:**
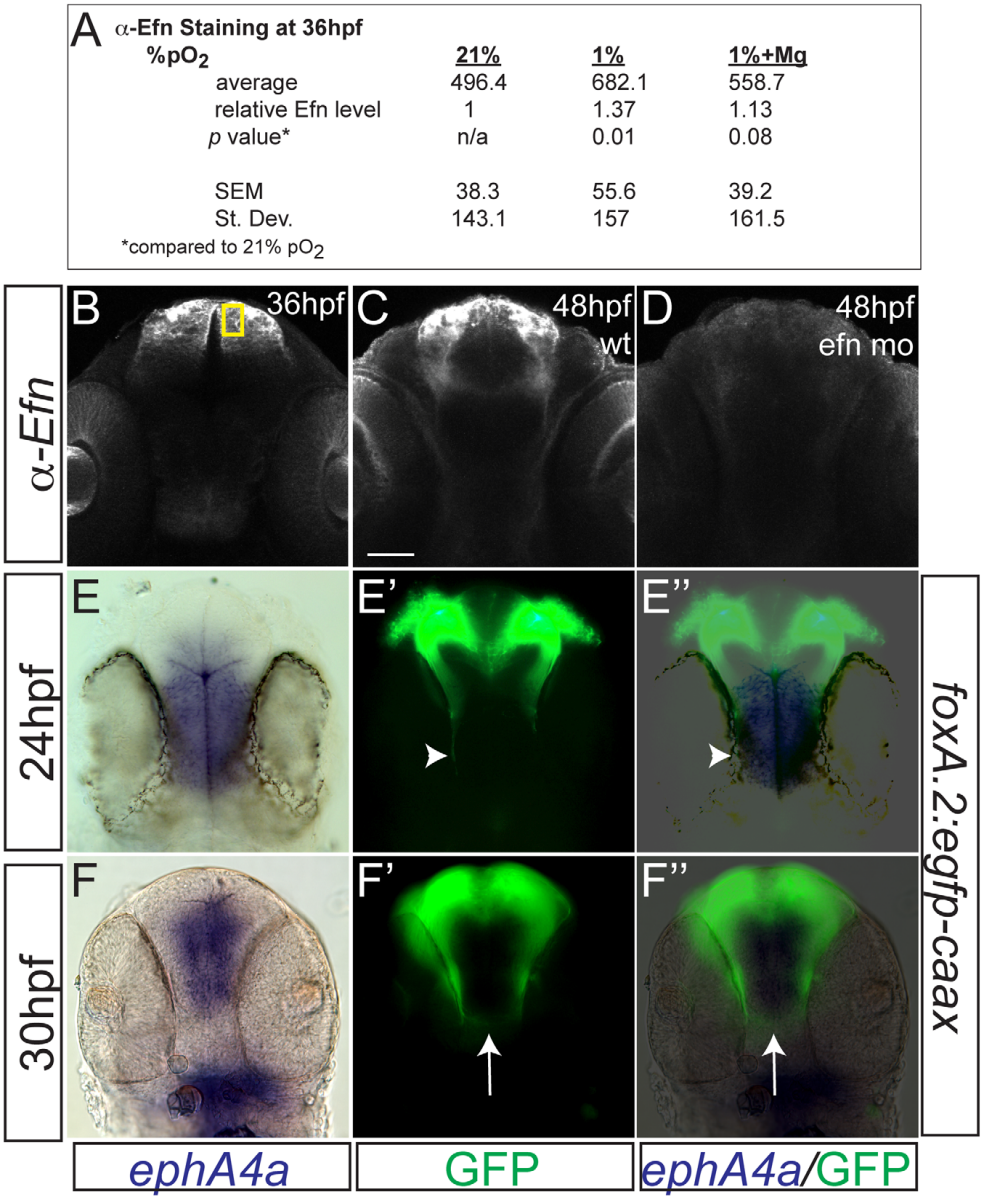
Hypoxia increased ephrinB2a expression in a pattern complementary to its receptor *ephA4a*. (A) Anti-EfnB2a intensity values at 36 hpf in the telencephalon, either following normoxia, or either hypoxia (1%), or hypoxia with 100 mM magnesium sulfate, from 24–36 hpf. (B) Confocal image of embryo at 36 hpf stained for ephrinB2, demonstrating the region of interest used to calculate α-ephrinB2 intensity. (C, D) Confocal images of embryos at 48 hpf stained for α-ephrinB2, demonstrating that *efn* morpholino (mo) effectively reduces ephrinB2a expression (D). (E–F″) Whole-mount double immunohistochemistry and in *situ*s for GFP and *ephA4a*, respectively, in Tg(*foxP2-A.2:caax*) embryos. (E–E″) During initial axon extension at 24 hpf, axons (arrowhead) travel along the edge of *ephA4a* expression domain. (F–F″) At 30 hpf, as TCPTc forms (arrow), the axons avoid the area of *ephA4a* expression.

TCPT neurons and axons express ephrinB2a during 24–36 hpf (Figure 5A), including in the TCPTc (Figure 5B). Hypoxia caused increased expression of ephrinB2a (Figure 5C–5E; Figure 6). Knock-down of *ephrinB2a* using a translation-blocking morpholino [33] led to loss of ephrinB2a expression (Figure 6A–6D), and protected against hypoxia pathfinding errors in the TCPT axons (Figure 5F, 5G). Further, injection of *ephrinB2a* morpholino into Tg(*elavl3:Gal4*); Tg(*foxP2-enhancerA.2:egfpcaax*); Tg(*UAS:hif1α* ^mut^ *-2A-TagRFP*) embryos led to rescue of TCPTc errors caused by misexpression of constitutively active *hif1α* ^mut^ (Figure 4K). These results suggest that *ephrinB2a* mediates some of the disruptive effects of hypoxia on pathfinding.

To determine how signaling by ephrinB2a is necessary for the pathfinding by TCPT axons, we expressed a form of ephrinB2a lacking the cytoplasmic signaling domain, *UAS*:*ephrinB2a(Δcytoplasmic*) [34] in TCPT neurons using Tg(*foxP2-enhancerA.2:Gal4*). Transient injections of *UAS*:*ephrinB2a(Δcytoplasmic)*, but not full-length *UAS*:*ephrinB2a*, prevented hypoxia-induced pathfinding errors (Figure 5H; Table 2). There was no effect on the TCPTc under normoxic conditions by expressing either truncated or full-length ephrinB2a (Table 2). This demonstrates that *ephrinB2a* lacking its cytoplasmic domain is able to decrease guidance errors cell-autonomously, perhaps by acting as a dominant negative to interfere with signaling coming from its “ligand” receptor-tyrosine kinase EphA4a or EphB4. This is consistent with *ephrinB2a* using a “reverse” signaling mechanism for TCPTc pathfinding [35].

We examined the pathfinding of TCPTc axons relative to the expression of *ephA4a* (Figure 6E–F″), a known receptor for *ephrinB2a*, that can act during midline commissure formation. We found that the TCPTc axons travel along the edge of the midline expressing *ephA4a* during the period of initial axon extension at 24 hpf (Figure 6E–6E″). As the TCPTc forms, the axons cross the midline, again avoiding the *ephA4a* expression domain (Figure 6F–6F″). These results suggest that the *ephrinB2a*-expressing TCPTc axons are responding to a signal from the *ephA4a*-expressing midline cells. Hypoxic up-regulation of *ephrinB2a* may disrupt the normal balance of signaling between the ligand/receptor pair of *ephrinB2a*/*ephA4a*, and thereby disrupt TCPTc formation.

### Magnesium protects against hypoxia pathfinding errors by downregulation of ephrinB2a

**Figure 7:**
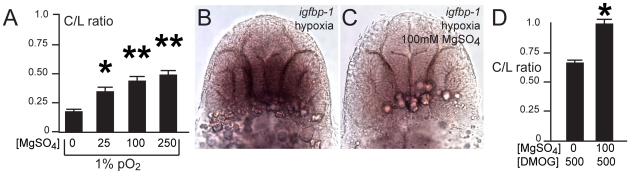
Magnesium sulfate administration protects against hypoxia-related axon pathfinding defects. (A) C/L intensity ratios of increasing magnesium concentrations (mM). *p<0.01; ** *p*<0.001. Error bars SEM. (B, C) Magnesium reduces activation of the *hif1α* pathway, as shown by decreased *igfbp-1* expression following hypoxia and concurrent magnesium exposure from 24–36 hpf. Bright-field images; ventral views, rostral top, scale bar 50 µm. (D) C/L intensity ratios shows rescue by magnesium of DMOG exposure from 24–36 hpf. *p<0.05. Error bars SEM.

Few neuroprotective agents have been demonstrated to affect neurodevelopmental outcomes of premature infants. Administration of magnesium sulfate to mothers of infants at risk for premature delivery improves neurodevelopmental outcomes and reduces rates of cerebral palsy [14], although the mechanism is unknown. We sought to determine whether magnesium could reduce pathfinding errors in hypoxic conditions. Embryos tolerated magnesium sulfate concentrations ranging from 25 to 250 mM during 24 to 36 hpf with no apparent morphologic defects, although above 250 mM there was approximately 30% mortality. Increasing concentrations of magnesium led to rescue of hypoxia TCPTc pathfinding errors (Figure 7A). We determined that magnesium can reduce activation of the *hif1* pathway, assayed by *in situ* for *igfbp-1* (Figure 7B, 7C). However, magnesium was unable to rescue commissure errors in embryos expressing activated *hif1α* ^mut^. Namely, in Tg(*elavl3:Gal4*); Tg(*foxP2-enhancerA.2:egfpcaax*); Tg(*UAS:hif1α* ^mut^ *-2A-TagRFP*) embryos, magnesium did not rescue pathfinding (Figure 4K). Magnesium did normalize Efn levels following hypoxia (Figure 6). Interestingly, magnesium was able to rescue pathfinding errors in the presence of DMOG (Figure 7D). These results show that magnesium does rescue the effects of hypoxia on pathfinding. Further, given the results with DMOG and *hif1α* ^mut^, it suggests that magnesium's primary mode of action might be through the prolyl hydroxylase or factor inhibiting hypoxia-inducible factor (FIH) pathways.

## Discussion

The increasing numbers of preterm births and associated adverse neurodevelopmental outcomes has highlighted the limited understanding of basic mechanisms underlying this problem [6]–[8]. Indirect radiological evidence in infants born prematurely shows altered CNS connectivity [9], [10], and neurodevelopmental disorders such as autism are associated with changes in axonal and synaptic gene expression [36].

We investigated whether hypoxia, a known complication of prematurity, can disrupt axon pathfinding. We found that hypoxia disrupts connectivity in the vertebrate CNS, leading to precise and reproducible errors in axon pathfinding. The disruption of pathfinding we observed is not due to apoptosis or broad effects on CNS development and specification. We have found that the pathfinding errors are due to hypoxia activation of the *hif1* pathway, and can be mimicked by chemical activators of *hif1*α, or by misexpression of a constitutively active form of *hif1*α. Further, the pathfinding errors are mediated by *ephrinB2a* reverse signaling, and can be rescued by knock-down of ephrinB2a. Finally, we have found that magnesium sulfate, which is used as a neuroprotective agent for impending preterm births, can reduce activation of the *hif1* pathway and decrease pathfinding errors following hypoxia.

While *hif1*α is broadly upregulated following hypoxia, we determined that activation of the *hif1* pathway disrupts pathfinding through a non-cell-autonomous effect. This may be due to hypoxia interfering with the balance of ligand-receptor signaling necessary for normal pathfinding. Thus, the same effect, disrupting axon guidance, can be achieved by at least one of two mechanisms: by increasing Eph receptor expression through *hif1* pathway activation in neighboring cells; or by increasing EphrinB2a expression in TCPT neurons. Usually, EphrinB2a acts as a ligand for one of the receptor tyrosine kinases (RTK) of the EphA3, EphA4, or EphB4 families, which in turn sets off an intracellular signaling cascade in the RTK-expressing cell [37]. However, EphrinB2 is also able to “reverse” signal, usually by tyrosine phosphorylation of its own cytoplasmic domain [35].

We found an evolutionary conservation of the molecular mechanisms mediating hypoxia's effects on pathfinding. Pocock and Hobert [13] described pathfinding defects in the invertebrate *C. elegans* caused by *hif1* pathway activation and increased expression of the Eph receptor VAB-1. Prior to our study there has only been indirect evidence showing disruption of axon pathfinding from hypoxia in vertebrates, and whether the same genetic pathways as in *C. elegans* would be involved was unknown. There is a marked increased in complexity of CNS structures, and increase in number of the members in gene families, from invertebrates to vertebrates. Thus, it is notable both that hypoxia can specifically disrupt vertebrate axon pathfinding, and that *ephrinB2a*, the closest zebrafish homolog to VAB-1, appears to be a primary mediator of *hif1* pathway activation.

In *C. elegans*, hypoxia leads to increased expression of the Eph receptor VAB-1 and aberrant crossing of the midline by PVQ interneurons and HSN motorneurons, a phenotype which can be rescued by knock-down of the ligand *efn-2* [13]. In contrast, we observed a failure of commissural axon crossing, even though we observed an up-regulation of the corresponding zebrafish ligand *ephrinB2a*. The reason for this difference between the two species, of increased crossing versus failure to cross the midline, is not clear, although there are several possible answers. First, it may reflect a bias in neurons assayed. Most of our analysis was done with an enhancer for axons that normally cross the midline. It is possible that with a different enhancer, for example of longitudinal axons, we might observe aberrant crossing. Second, some of the *C. elegans* neurons affected by hypoxia are not strictly ipsilateral in their normal projections. The PVQ-L crosses the “midline” to travel with the PVQ-R axon before switching back to the left side, while the HSN motorneurons project through the nerve ring to the opposite side of the nervous system [38]. Thus, the crossing of the midline may reflect a disorganization of pathfinding, rather than a loss of signals preventing midline crossing. Third, there are significant differences in midline guidance in the nervous systems of zebrafish and *C. elegans*. The *C. elegans* midline is defined by a guidepost function of midline motor neurons [39], whereas in zebrafish and other vertebrates glial bridges provide some of this role [40]. VAB-1 over-expression in *C. elegans* is able to produce axon guidance defects [41], so perhaps differences in the midline structures of the nervous systems results in different expression patterns and usage of the eph/ephrin signaling pairs.

We found that hypoxia caused increased expression of ephrinB2a by hypoxia, and subsequent failure of TCPTc axons to cross the midline. In mammals, ephB and ephA receptor family members, and ephrinB ligands, guide development of the corpus callosum, by a complex series of actions including effects on midline glial growth, on modulating axon responsiveness, and on reverse signaling in axons as they cross the midline [42], [43]. In zebrafish, the ligand *ephrinB2a* is expressed in telencephalic neurons and their axons as they extend towards the midline [33, this study], and we have found that the receptor *ephA4a* is expressed in the midline of the CNS during axon extension. These results support a model where signaling between *ephrinB2*-expressing axons and *ephA4a*-expressing midline cells helps guide the axons as they cross the midline. Alterations in levels of ligand or receptor, for example by hypoxia-induced up-regulation of *ephrinB2*, could thus impair normal TCPTc formation.

Comparing degree of hypoxia in different animal species is difficult, and extrapolating 1% hypoxia (1% pO_2_) in zebrafish to equivalent effects in premature infants is not straightforward. Since zebrafish can survive extended periods of hypoxia and even anoxia [this study]; [ 15,16], 1% hypoxia might be equivalent to relatively “mild” hypoxia in human infants. Premature infants at 36 weeks gestation have been found to spend more than 8 hours/day at less than 90% oxygen saturation [12], and infants with chronic lung disease or congenital heart disease have more significant decreases in oxygenation. Finally, using 1% hypoxia to examine the mechanisms underlying hypoxia's effect allowed us to examine the genetic pathways and more noticeable effects on CNS pathfinding. It seems likely that less stringent hypoxia would have similar, but less marked effects. Similarly, since typical human serum magnesium levels are ∼1 mM, the human physiological corollary to bathing zebrafish in 250 mM magnesium sulfate is unclear. However, the current magnesium dose given to expectant mothers is a single bolus dose of 4 g, followed by a continuous infusion of 2 g/hour until delivery [14]. Further, it is not known what fetal magnesium levels are following the dose, and whether there are differences in neurodevelopmental outcomes depending on overall dose or post-natal levels. While there are obvious limitations to extrapolating our findings to human infants, it is possible that increasing magnesium levels in human infants could have protective effects [44]. For these reasons we think that the mechanisms and genetic pathways activated by hypoxia in zebrafish may also be relevant in human development.

In addition to conservation of molecular mechanisms, is there conservation of effects of hypoxia on specific subsets of axon pathfinding in humans? Our work found that only a subset of axons was affected by hypoxia. For example, using our transgenic reporter line, we found that commissural axons extending from forebrain neurons were disrupted, whereas we did not observe problems in the longitudinal axons or in the optic chiasm. In humans, prematurity has been shown to affect connectivity of commissural structures, including the corpus callosum [10], as well as non-commissural axon tracts such as the internal capsule, superior fasciculus, uncinate fasciculus, and external capsule [10], [45]. These changes in connectivity are correlated with decreased overall intelligence quotient (IQ) score [46], with particular correlations noted between corpus callosum disruption and IQ as well as attention-deficit hyperactivity disorder [47], [48]. In addition, autism is associated with prematurity and disruptions of both intra-hemispheric and inter-hemispheric connectivity [49], [50]. A significant caveat to these studies, however, is the indirect nature of the measurements, relying upon fractional anisotropy based on water diffusion measured by magnetic resonance imaging, and that differences in cortical volume and/or synaptic changes could also impact the clinical findings.

This study raises several issues for future study. First, we found considerable variability in the degree of disruption of axon pathfinding from animal to animal. Following hypoxia, some animals had no TCPT commissural axons cross the midline, while in other animals the TCPTc was hardly or not affected. This suggests that other homeostatic mechanisms, as yet unknown, may help prevent or ameliorate the pathfinding errors. A second question concerns why certain axons (and neurons) are particularly susceptible to hypoxia. While we were found that the TCPTc was affected following hypoxia, in other axon groups, for example the optic tracts, we never observed pathfinding errors following hypoxia. Is this due to some intrinsic feature of different neuron types, or because certain molecules, such as *ephrinB2a*, are expressed only in certain neurons? Since we did observe errors in other axon tracts when we used pan-axonal immunohistochemistry, it is likely that a wider subset of neurons and axon tracts are affected. Third, does hypoxia affect the development of the other main determinant of connectivity, namely synapses? Microarray data in rodents has shown that hypoxia causes altered expression of multiple synaptic genes [51]. Additional studies will need to characterize effects of hypoxia on synapse development. Fourth, magnesium was protective against the hypoxic pathfinding disruption. Magnesium sulfate is currently used as a single-dose agent immediately prior to preterm births [14], but future work could examine whether elevated magnesium levels are associated with improved neurodevelopmental outcomes, as well as what the molecular target of magnesium is.

Our finding of disrupted connectivity in the brain following hypoxia and the involvement of a conserved genetic pathway suggests one mechanism that may contribute to the diverse neurodevelopmental impairments seen in premature infants. Premature infants have elevated rates of attention-deficit disorder, autism, cerebral palsy, epilepsy, psychiatric disorders, and cognitive impairment [2]–[5], and perhaps disruptions of connectivity might be responsible for some of these outcomes. Although cognitive impairment is the most common of these chronic neurodevelopmental problems [4], it is not known, for example, why certain infants develop autism as opposed to attention-deficit disorder, or what characteristics of the hypoxia lead to different clinical outcomes. The identification of the precise effects and molecular mediators of hypoxic injury in the developing vertebrate brain offers the possibility for improved understanding and novel therapeutic approaches.

## Materials and Methods

### Ethics statement

All zebrafish experiments were performed with supervision and in strict accordance of guidelines from the University of Utah Institutional Animal Care and Use Committee (IACUC), regulated under federal law (the Animal Welfare Act and Public Health Services Regulation Act) by the U.S. Department of Agriculture (USDA) and the Office of Laboratory Animal Welfare at the NIH, and accredited by the Association for Assessment and Accreditation of Laboratory Care International (AAALAC).

### Fish stocks and embryo raising

Adult fish were bred according to standard methods. Embryos were raised at 28.5°C in E3 embryo medium and staged by time and morphology [52]. For *in situ* staining and immunohistochemistry, embryos were fixed in 4% paraformaldehyde (PFA) in PBS overnight (O/N) at 4°C, washed briefly in PBS with 0.1% Tween-20, dehydrated, and stored in 100% MeOH at −20°C until use.

Transgenic fish lines and alleles used in this paper were the following: Tg(*foxP2-enhancerA.2:egfp-caax*)^zc69^; Tg(*foxP2-enhancerA.2:Gal4-VP16_413–470_*)^zc72^; Tg(*elavl3:Gal4-VP16_413–470_*); Tg(*UAS:hif1*α *-2A-egfpcaax*)^zc73^; Tg(*UAS:hif1*α *-2A-TagRFP*)^zc74^; Tg(*UAS: hif1α* ^mut^ *-2A-egfpcaax*)^zc75^; Tg(*UAS: hif1α* ^mut^ *-2A-TagRFP*)^zc76^, where 2A is a viral hydrolase peptide sequence [29]; and Tg(*otpb.A:Gal4-VP16_413–470_*, *myl7:EGFP*)^zc57^ [30]. Injection of DNA constructs and generation of stable transgenic lines was performed essentially as described [17]. Lines are available upon request.

### Hypoxia reagents

To induce hypoxia, embryonic zebrafish were placed in a sealed plexiglass chamber connected via a controller that monitored and adjusted nitrogen gas flow to a desired pO_2_ set point (Biospherix Ltd.). We observed that equilibration of oxygen partial pressures in water could take several hours measured with a dissolved oxygen water meter (Control Company). Therefore we pre-equilibrated all solutions to either normoxia or hypoxia for at least 4 hours before use, and transferred embryos into and out of pre-equilibrated solutions. At the desired time, embryos were placed into media that had been equilibrated to the hypoxic conditions. To terminate hypoxia, embryos were returned to media kept in normoxic conditions. Morphological staging was used to help determine age at fixation for analyses.

### Scoring TCPT axon errors, C/L intensity ratios

To assay whether a TCPT pathway had decreased commissural crossing, we measured the total fluorescence intensity (average intensity *x* area) of a rectangular area placed over the commissure or longitudinal tract. A confocal z-stack was taken of the region using identical confocal settings (20× objective, laser power 10%, gain 1.25%, offset 2%, PMT 400, speed 2.5 µs/pixel). We used ImageJ to calculate an average intensity projection of 10 slices (step size 2.8 µm), then measured total fluorescence intensity in a rectangle of set size (0.12×0.25; 10 µm×30 µm) over the midline of the commissural TCPT pathway or over the longitudinal axons prior to their decussation into the TCPT. A ratio of the commissural vs. longitudinal axon intensity was calculated (C/L ratio) (Figure 1F). Some experimental variation was noted, and so results were only directly compared for experiments performed on the same day. For determination of C/L ratios of hypoxia effects at different ages (Figure 1G), 24 or more embryos were imaged per age.

### Immunohistochemistry and *in situ* hybridization

Immunohistochemistry was performed as previously described [17], [30]. Antibodies used were: mouse anti-acetylated tubulin 1∶250, rabbit polyclonal anti-tyrosine hydroxylase 1∶400 (Millipore), mouse monoclonal anti-GFP 1∶250 (Millipore), goat polyclonal anti-EphrinB2a 1∶20 (R&D Biosystems), rabbit anti-phosphohistone H3 1∶500 (pH 3 polyclonal, Upstate Biotechnology), Cy-3 anti-rabbit 1∶400, Alexa 488 donkey anti-mouse 1∶400 (Invitrogen), Alexa 555 rabbit anti-goat 1∶100 (Invitrogen).

Double immunohistochemistry for GFP and EfnB2a was performed by permeabilization using 10 µg/ml Proteinase K in PBST, re-fixation for 10′ with 4% PFA, further permeabilization with 0.1M citrate and 0.1% Triton in PBS, incubation with mouse anti-GFP and goat anti-ephrinB2a, followed by washing with PBST/1% DMSO and incubation with donkey anti-mouse Alexa 488 and rabbit anti-goat Alexa 555.

Whole-mount *in situ* labeling for *igfbp-1* was performed using an anti-sense probe generated by NotI digestion and transcription using T3 polymerase from pCR4 Blunt-ZF IGFBP-1 [22], as previously described [53].

### Cloning and transgenic fish lines

Expression clones were built using the Tol2 kit [54] and recombination reactions with Gateway (Invitrogen) plasmids. For clones lacking an expressed fluorescent marker, either a *cmlc2*:EGFP (official nomenclature *myl7*:EGFP) or *cmlc2*:TagRFP transgenesis marker was used in the final construct. The identity of constructs was confirmed by restriction enzyme digests and by sequencing on both strands (for coding sequences) or by partial end-sequencing (for enhancers). Specific plasmids used for cloning were p5E-foxP2-enhancerA.2; p5E-*elavl3*; p5E-10xUAS; pME-basEGFP-caax (middle entry clone with EGFP-caax preceded by minimal promoter); pME- basGal4-VP16_413–470_; pME- *hif1*α (no stop) or pME- *hif1α* ^mut^ (no stop); p3E-pA; p3E-2A-eGFPcaax-pA; p3E-2A-TagRFP-pA; into either pDestTol2pA2, or pDestTol2CG2/pDestTol2CR3 (pDestTol2pA3 with *cmlc2*:EGFP or *cmlc2*:TagRFP transgenesis marker, respectively) [17], [29], [30], [54].

We cloned and sequenced the zebrafish *hif1*α cDNA (*hif1α*β). We generated a constitutively active form of *hif1*α, *hif1α* ^mut^, by mutating proline 621 to alanine in the conserved LXXLAP motif on HIF-1 [28]. This mutation prevents the hydroxylation of HIF-1 and thereby its subsequent proteosomal degradation. Zebrafish *hif1*α was cloned based on the GenBank sequence AY326951, adding attB1F and attB2R sequences for cloning using the Gateway system (Invitrogen): forward primer: bp 230–250 (*hif1*α sequence italicized, attB1F regular type, start codon underlined) 5′-GGGGACAAGTTTGTACAAAAAAGCAGGCT*ACCCAGGAATGGATACTGGAG*-3′; reverse primer bp 2587–2606 5′-GGGGACCACTTTGTACAAGAAAGCTGGGT*GGAAGAGTGTCCGCAGTTGC*-3′. PCR was performed using Tuebingen wild-type cDNA, and the resulting 2.4 kb fragment was recombined into pDONR221 and sequenced. To generate the *hif1α* ^mut^construct, the proline at position 557 was changed to alanine with site-directed mutagenesis by overlap PCR (**C**CT to **G**CT at nucleotide 1677) and the final clone was confirmed by full-length sequencing. C-terminal fusion constructs of *hif1*α were made by overlap PCR changing the stop codon (TGA) to glycine (GGA).

### Microscopy and image analysis

Image acquisition and analysis were performed as described previously [17]. Immunostained embryos were transferred step-wise into 80% glycerol/20% PBST, mounted on a glass slide with a #0 coverslip placed over a well made using electrical tape, and imaged on a confocal microscope. Confocal stacks were projected in ImageJ, and images composed with Adobe Photoshop and Illustrator. For imaging fluorescent immunohistochemistry and bright-field *in situ* staining for Figure 6 E″ and F″, the “Exclusion” function on Adobe Photoshop was used to combine the layers, with the “Exposure” set at −4.0 for the bright-field image.

Determination of α-Efn staining intensity was calculated using identical confocal settings for imaging all of the embryos (laser power 10%, gain 1.25%, offset 2%, PMT 400, speed 2.5 µs/pixel). Intensities were calculated in a rectangle of set size (0.12×0.25; 10 µm×30 µm) over the midline of the telencephalon using maximum intensity projections of 5 z-slices (step size 2.8 µm) (Figure 6B).

### Morpholino injections

One-cell stage embryos were injected with 1 nl of 3 ng/nl EfnB2a translation-blocking morpholino [33] as previously described [17].

### Acridine orange staining

Live control and hypoxic embryos (72 hpf) were collected into 1.5 mL tubes, stained for 30′ in 5 µg/mL acridine orange in E3 embryo medium, then washed for 30′ at RT in E3 on a nutator. Embryos were then anesthetized and mounted in low-melt agarose containing tricaine. Live imaging was performed using a confocal microscope (PMT 581V, scan size 640×480, speed 1.79 s/scan, UPLFL 20× objective, z-step size 2.8 µm, laser intensity 15.0%)

### Scoring apoptosis/acridine orange staining

ImageJ was used to score 20 z-slices (step size 2.8 µm) counting dorsally form the equator of the lens. A square with an area of 100 µm^2^ was drawn with its top boundary immediately caudal to the olfactory pits, sides immediately medial to the eyes, and lower boundary at the center (rostral to caudal axis) of the lens (Figure 3E, E′). Apoptotic cells were counted that lay within the square including those that contacted the boundaries.

### Drug and Mg^2+^ treatment

Embryos were manually dechorionated prior to 24 hpf. For Mg^2+^ treatment, E3/PTU-containing solution was mixed with the appropriate concentration of magnesium sulfate (25–250 mM); for dimethyloxalylglycine (DMOG) (Sigma) or CAY10585 (Cayman Chemical), the drug was diluted in 0.1% DMSO and added to E3/PTU to make final concentrations of 100–750 µM (DMOG) or 20 µM (CAY10585). Embryos were incubated during an exposure period from 24–36 hpf, and at 36 hpf embryos were returned to E3/PTU. Fixation with 4% PFA occurred at 72 hpf.

## Supporting Information

Figure S1 Expression of activated *hif1α* ^mut^ near TCPT axons does not disrupt axon pathfinding. Confocal whole-mount images, double anti-GFP and anti-RFP immunohistochemistry, of 72 hpf embryo Tg(*foxP2-A.2:caax*), Tg(*otpb.A:Gal4-VP16*), injected with *UAS:hif1α* ^mut^ *-2A-TagRFP*, ventral views, rostral top, maximum intensity projections, scale bars 50 µm. Arrow points to axons expressing *hif1α^mut^* near the TCPT.
(TIF)
