# Supplementary figures and images for: Hypoxia Disruption of Vertebrate CNS Pathfinding through EphrinB2 Is Rescued by Magnesium
Source: PLoS Genet. 2012 Apr 12;8(4):e1002638. doi: 10.1371/journal.pgen.1002638 (PMC3325188; doi:10.1371/journal.pgen.1002638)

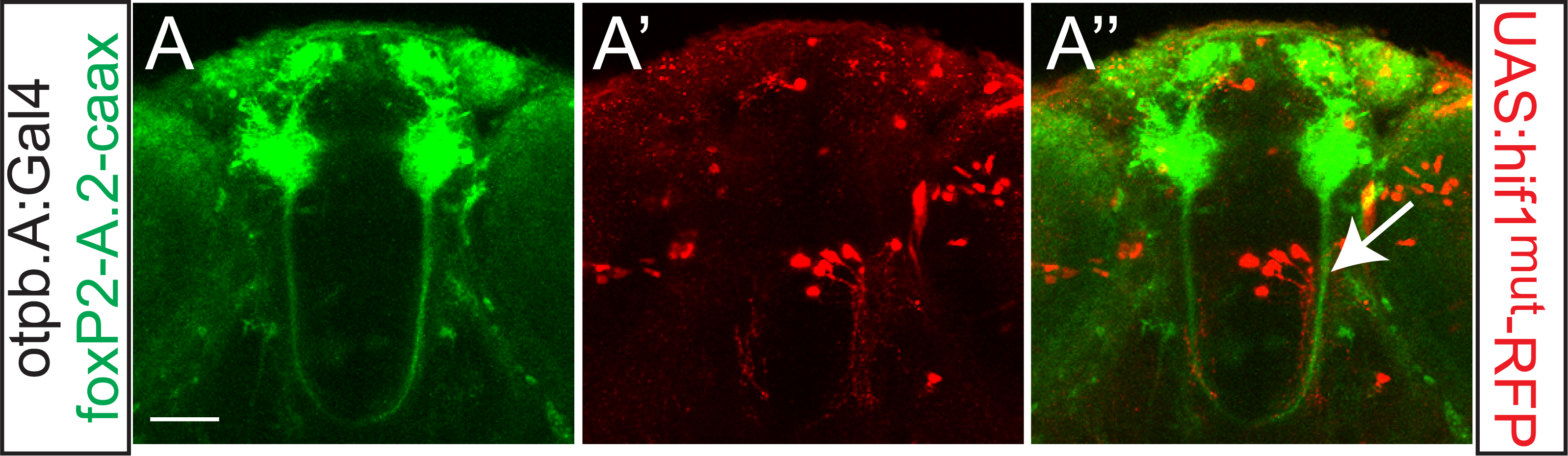

Supplement: Figure S1 — Expression of activated hif1α mut near TCPT axons does not disrupt axon pathfinding. Confocal whole-mount images, double anti-GFP and anti-RFP immunohistochemistry, of 72 hpf embryo Tg(foxP2-A.2:caax), Tg(otpb.A:Gal4-VP16), injected with UAS:hif1α mut -2A-TagRFP, ventral views, rostral top, maximum intensity projections, scale bars 50 µm. Arrow points to axons expressing hif1αmut near the TCPT. (TIF) [file pgen.1002638.s001.tif]
